# A Laboratory Study on Non-Invasive Soil Water Content Estimation Using Capacitive Based Sensors

**DOI:** 10.3390/s19030651

**Published:** 2019-02-05

**Authors:** Amir Orangi, Guillermo A. Narsilio, Dongryeol Ryu

**Affiliations:** Department of Infrastructure Engineering, School of Engineering, The University of Melbourne, Parkville, VIC 3010, Australia; amir.orangi@unimelb.edu.au (A.O.); dryu@unimelb.edu.au (D.R.)

**Keywords:** agriculture, capacitive sensors, dielectric constant, remote sensing, surface soil water content

## Abstract

Soil water content is an important parameter in many engineering, agricultural and environmental applications. In practice, there exists a need to measure this parameter rather frequently in both time and space. However, common measurement techniques are typically invasive, time-consuming and labour-intensive, or rely on potentially risky (although highly regulated) nuclear-based methods, making frequent measurements of soil water content impractical. Here we investigate in the laboratory the effectiveness of four new low-cost non-invasive sensors to estimate the soil water content of a range of soil types. While the results of each of the four sensors are promising, one of the sensors, herein called the “AOGAN” sensor, exhibits superior performance, as it was designed based on combining the best geometrical and electronic features of the other three sensors. The performance of the sensors is, however, influenced by the quality of the sensor-soil coupling and the soil surface roughness. Accuracy was found to be within 5% of volumetric water content, considered sufficient to enable higher spatiotemporal resolution contrast for mapping of soil water content.

## 1. Introduction

Soil water content is a parameter with implications in an array of engineering, hydrology, climate science, water resource management, remote sensing and agricultural applications [1,2,3,4,5,6]. The challenge of increasing water use in agriculture, which is known to be the largest consumer of water resources (e.g., see [7]), can be alleviated by better-informed irrigation decisions and smart farming systems that are based on accurate measurements of soil water content [8,9,10]. In addition, accurate and rapid measurements of soil water content can enhance site assessments in a broad range of civil engineering applications such as road construction, since the soil moisture is an important parameter to derive the strength and the integrity of the infrastructure [11]. Furthermore, in bushfire management, the fuel availability estimates used for issuing warnings are partly based on the soil moisture deficit [12].

Surface soil moisture comprises only 0.05% of the Earth’s total fresh water. Although this value is small, the amount of soil moisture is imperative in agriculture for crop development, irrigation management, crop type selection and plant stress [13,14,15,16]. Additionally, spatiotemporal variations of near surface soil water content are of paramount significance in a number of applications due to its very large inhomogeneity [17,18,19]. As such, the near surface soil water content is an integral hydrological and meteorological parameter for ground truthing remotely sensed data, mapping variable sources of streamflow, developing large scale surface water and energy balance models and improving the land component of climate models, including global circulation models [17,20,21,22,23,24,25,26]. In precision agriculture, it is the soil water content in the root zone, not the near surface water content, that determines the amount of water available to a plant. However, the water available to the plant often can be inferred from near surface soil water content information (e.g., see [19,27,28,29,30]). Nonetheless, with the current soil water content measurement techniques measuring the surface soil water content become difficult to assess its spatiotemporal variability [31,32].

Soil water content can be directly measured using the oven drying method which is accurate and inexpensive; however, it is time-consuming and labour-intensive. In addition, there are indirect techniques which utilise other soil parameters as a proxy to estimate soil water content. Neutron probes are commonly used for these indirect techniques; however, there are limitations associated with their use. These limitations are primarily due to the probes containing radioactive materials and include the high cost of equipment, the requirement of a certificate to operate, the inability to use as a continuous monitoring tool and unreliability to estimate near surface soil water content [2,20,33]. Furthermore, the common methods of measuring soil water content often cannot provide immediate feedback [34]. The disadvantages of traditional soil water measurement methods associated with time and cost are exacerbated by the large spatial extent of measurements required for irrigation management in agriculture and motivate the development of cost-effective and non-invasive alternatives [35,36]. 

Alternative techniques which address some of the limitations of traditional methods include dielectric methods. In these, the electrical properties of soil are utilised as a proxy to estimate soil water content. For example, Time Domain Reflectometry (TDR) probes, Frequency Domain Reflectometry (FDR) probes, capacitive probes, impedance probes, Ground Penetration Radar (GPR) and Electromagnetic conductivity (EM) antennas have been used to estimate water content in various applications (e.g., see [10,37,38,39,40,41]). However, the accessibility of these methods is limited by the high cost of equipment and difficult result interpretation. Furthermore, despite the non-invasive nature of GPR and EM antennas, the requirement of probe insertion into the soil makes the TDR, FDR, capacitive and impedance probes labour intensive, particularly for hard or dense soils. Additionally, by being invasive, repeated measurements at the same location can make the measurements unreliable [42]. To compensate for the high cost involved in some of the aforementioned methods, there exist other common low-cost sensors which, although invasive, demonstrate good performance. The heat pulse soil moisture sensors using single or dual probe designs have been introduced in this context [43,44,45,46,47,48,49,50]. Furthermore, recent developments in capacitive soil moisture sensors have enabled low cost means in soil water content measurement [49,50]. Nonetheless, they have not eliminated the need for probe insertion. For this purpose, a needle-free heat pulse sensor system has recently been developed [51]. Although this sensor has no needle and is inexpensive, burying it in the soil causes soil disturbance [51]. None of these developments, however, have focused on non-invasive measurements of near surface soil water content. Instead, remote sensing applications have been widely used as the primary source of information of surface soil water content. However, they often lack the required resolution for certain applications [31,52]. It has been suggested that understanding the sub-footprint scale of the variability of remotely sensed soil water content is an important factor to fully utilise these data [31]. 

Motivated by the importance of soil water content and the drawbacks of its current measurement techniques, particularly for near surface soil water content, this research aims to develop a new non-invasive, low cost and capacitive-based technique for estimating near surface soil water content. We hypothesise that given the accuracy of the relationship between soil water content and its dielectric properties, which is widely known, the surface soil water content can be estimated from the surface using a non-invasive capacitive sensor (since capacitance is linked to the dielectric constant and the sensor geometry). To evaluate this hypothesis, we first compared three new non-invasive capacitive sensors developed to estimate soil volumetric water content and we examined their performance for four different soil types and for a range of water contents. Subsequently, based on the comparative performance of these three sensors, a fourth sensor was designed and manufactured to substantially reduce the limitations of the previous versions. The sensors, particularly the fourth one, demonstrated great potential in detecting variation in soil water content from the ground surface. Moreover, it is concluded that the soil-sensor coupling and roughness of the soil sample surface play an important influencing role in the performance of the sensors. 

To address the objective of this research, this manuscript is organised as follows: In Section 2, an overview of dielectric permittivity and soil moisture is introduced. Section 3 and Section 4 comprise descriptions of the materials and methodologies used. The results, analyses and discussion are presented in Section 5. In Section 6, we discuss potential practical applications of the sensors as well as the limitations of the research. Finally, summaries of the findings and recommendations for future work are presented in Section 7. 

## 2. Theory and Background: Dielectric Permittivity and Soil Moisture

Current non-destructive soil water content estimation techniques such as GPR and TDR are based on measuring the dielectric permittivity of soil. The dielectric permittivity, ε (F/m), is a complex number which measures the degree to which a material is polarised when it is subjected to an electrical field and it can be represented as shown by [41]:

(1) $$\varepsilon =\varepsilon^{\prime }-j\varepsilon^{\prime \prime }$$

where $\varepsilon^{\prime }$ is the real component of the dielectric permittivity (F/m), $j=\sqrt{-1}$ is the imaginary number and $\varepsilon^{\prime \prime }$ is the imaginary component of the dielectric permittivity (F/m) known as the dielectric loss. The ratio between a material’s dielectric permittivity and that of air (ε_o_ ≈ 8.85 × 10^−12^ F/m) is known as the relative dielectric permittivity, κ and can be written as:

(2) $$\kappa =\frac{\varepsilon \;}{\varepsilon_{o}}=\kappa^{\prime }-j\kappa^{\prime \prime }$$

where κ’ is known as the dielectric constant and κ’’ is known as the loss factor. In an unsaturated soil, water has the highest dielectric constant (κ’ ≅ 80), which is noticeably larger than the dielectric constant of minerals (2 < κ’ < 7) and of air (κ’ = 1). The bulk dielectric permittivity of soil, a mixture of these three elements is, therefore, influenced mostly by the water content. Indeed, a strong correlation between the soil volumetric water content and the (real) dielectric constant is reported in the literature. There are several studies which investigated the correlation between soil volumetric water content and its real dielectric constant, considering parameters such as soil type, salinity, density, temperature and frequency of measurements (e.g., [53,54,55,56,57]). Within this context, Topp, Davis and Annan [53] performed TDR measurements on four types of soil to propose what it is today the most commonly used empirical model: for a low-loss homogenous material (i.e., low or negligible$\;\kappa^{\prime \prime }$), the correlation between the apparent dielectric constant, $\kappa^{\prime }$ and its volumetric water content, θ, is:

(3) $$\theta =4.3\times 10^{-6}{\;\kappa^{\prime }}^{3}-0.00055{\;\kappa^{\prime }}^{2}+0.0292\kappa^{\prime }-0.053$$

Further, capacitance, C, is the ability of a material to store an electrical charge. The capacitance of a capacitor is related to the dielectric constant of the dielectric material used as the insulator [58] such that:

(4) $$C={\kappa g\varepsilon }_{0}$$

where κ is the relative dielectric permittivity, g is a geometric constant and $\varepsilon_{0}$ is the permittivity of a vacuum (F/m). Measurement of soil permittivity through capacitance methods was first introduced by Dean, et al. [59], who developed a capacitance sensor operating at a frequency of 150 MHz for the purpose of creating a cost-effective and safe in situ method [58]. Furthermore, due to their relatively low cost and ease of operation, capacitive sensors are becoming increasingly popular among researchers and practitioners [60]. Most importantly, the relationship between water content and the dielectric constant of a soil is widely accepted to be accurate [42,58]. We compare four new capacitive sensors developed to non-invasively estimate the soil volumetric water content by utilising the relationship between the volumetric soil water content and the dielectric constant (Equation (3)) and the relationship between the capacitance and the dielectric constant (Equation (4)). 

## 3. Materials and Methods

This section describes the experimental framework including the material and methods used to address the objectives of this research.

### 3.1. Tested Soils 

Four different soil types collected from Victoria, Australia, whose characteristics are summarised in Table 1, are used for testing. Soils are selected to cover a range of grain sizes and textures based on grain size distribution analysis according to Australian Standards [61,62]. In addition, the plasticity index (Plastic Limit, PL and Liquid Limit, LL) were determined following Australian Standards [63,64]. The Organic Matter (OM) was measured using the Loss on Ignition (LOI) method described in the American Society for Testing and Materials (ASTM) standards [65]. The salinity of the samples was estimated using the conductivity, σ, of the samples at saturation point ($\sigma =\;\kappa^{\prime \prime }\;\cdot \;\omega \cdot \;\varepsilon_{0}\;$), where $\kappa^{\prime \prime }\;$is the loss factor (see Equation (2)) and ω is the angular frequency, as proposed by Santamarina and Fam as well as Narsilio, et al. [66,67]. 

Based on the unified soil classification system (USCS) described in the Australian Geotechnical Site Investigations standard [68], the first soil is classified as a poorly graded sand (SP) denoted in this manuscript (and locally known) as Brighton Group Sand; the second soil as a fine-grained silty sand (SM) referred to as Silty Sand in this manuscript; the third soil as a low to medium plasticity silty clay sample (CL) denoted as Camb Clay in this manuscript and the fourth sample is classified as a low to medium plasticity clayey silt (ML) referred to as Bun Silt in this manuscript. These clay samples belong to the Silurian Melbourne geological formation, which contains primarily illite and kaolinite minerals. Based on the estimated values of salinity, the Brighton Group Sand and the Bun Silt are considered as non-saline soils whereas the Silty Sand and Camb Clay samples are considered as moderately saline according to Agriculture Victoria [69]. 

### 3.2. Dielectric Probe: Benchmark Dielectric Measurements

The complex dielectric properties of soil samples can be measured by means of an open-ended coaxial line technique [70]. In this work, a 2.2 mm diameter coaxial slim form Agilent dielectric probe (Keysight Technologies, Santa Rosa, CA, USA) (with a 0.51 mm diameter centre conductor and a 1.68 mm diameter insulator) connected to an N9923A FieldFox Vector Network Analyser (VNA) (Keysight Technologies), was utilised to measure the complex dielectric properties of the soils at different frequencies. These measurements help in evaluating the performance of the capacitive sensors. The slim form probe is reported in the literature to have been used to investigate the relationships between soil dielectric properties and other parameters such as water content, thermal conductivity, temperature, frequency and pH [71,72,73]. The complex scattering parameter of the material under test is measured and subsequently converted to the complex dielectric permittivity by means of proprietary software [74]. All the experiments were conducted in a controlled laboratory environment with a constant temperature range of 19 to 21 degrees Celsius.

### 3.3. Capacitive Sensors

**Figure 1:**
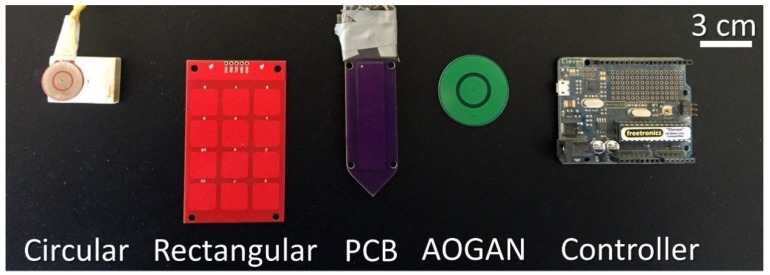
Capacitive sensors: Circular, Rectangular, PCB Gadget (PCB) and AOGAN sensors (left to right). The Arduino-based board controller is also shown to the right.

The four capacitive sensors used in this study are depicted in Figure 1 alongside an Arduino-based board use as a controller board. The sensors are (1) an AD7746 sensor, denoted as “Circular,” (2) an MPR121 sensor, denoted as “Rectangular,” (3) a PCB Gadget sensor, denoted as “PCB” and (4) a newly designed and built sensor denoted as “AOGAN.” The sensors are Capacitance-to-Digital Converters (CDC). The Circular and Rectangular sensors are typically used as keypads; however, in this work, they were programmed to measure the capacitance of the soil samples. The Circular, Rectangular and AOGAN sensors were connected to an Arduino-based board (Freetronics, Croydon South, VIC, Australia) and utilised a C++ platform to communicate and transmit the measured capacitance values. Similarly, the PCB sensor transmitted the capacitance reading through a USB cable to a CoolTerm computer program (provided by PCB Gadget) without the need of an Arduino-based controller board.

Regarding sensor specifications, the Circular sensor is composed of two concentric plates comprising the electrodes. This sensor can measure up to 24 pF capacitance, with a linearity of ± 0.01% and accuracy of ± 4 fF factory calibrated. The positive supply voltage can vary between + 0.3 and + 6.5 V and it has an operational frequency of approximately 32 kHz [75]. The Rectangular sensor has 12 capacitance sensing inputs and was programmed to measure a capacitance range from 0.45 pF to over 340 pF (depending on the programming code), has a positive supply voltage of 1.71 to 3.6 V operated at 400 kHz [76] and the sensing electrodes are covered with an insulating layer. The PCB sensor is a capacitive sensor comprising a single electrode to measure changes in the capacitance of a material operating at a frequency of 500 kHz [77]. The fourth sensor, the AOGAN sensor, was manufactured by adopting a similar shape to the Circular sensor, with an insulating layer, with a similar controller board as the Rectangular sensor and with an operating frequency of 400 kHz. The sensor was also operated by an Arduino-based board.

## 4. Experimental Procedure

A description of the testing and development of the sensors for the non-invasive soil water content estimation is presented in the following sections. 

### 4.1. Sample Preparation and Dielectric Measurements 

**Figure 2:**
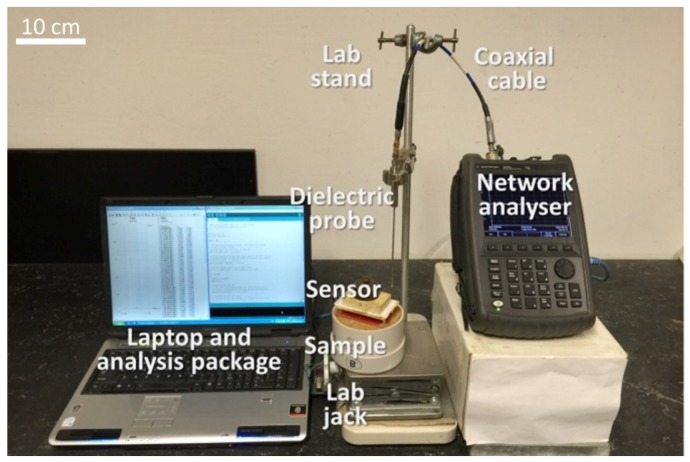
Typical experimental setup.

Soil samples were crushed and subsequently prepared from the air-dry condition to saturation, by incrementally adding deionised water. This incremental addition of water was achieved by thoroughly mixing the soil and allowing adequate curing time for the samples to attain a homogeneous state. Deionised water was used to minimise the introduction of any foreign ions to the soil samples, which may have potentially influenced the dielectric properties. The soil was then transferred to a non-dielectric (PVC) container with a known volume and a size adequate to accommodate the Agilent dielectric probe and capacitive sensor. We initially used a PVC container with similar dimensions to a standard compaction mould (approximate volume of 1000 cm^3^, see [78]) and later changed to a smaller PVC mould with the same diameter (10 cm) and a volume of approximately 160 cm^2^. We initially tested the bulk density effects, by preparing samples at different dry densities using the larger mould. However, since the sensitivity of the sensor to density variation was deemed to be insignificant, we opted to use a smaller mould to expedite the experimental program. It is important to note that the size of the smaller PVC mould was selected such a way that the thickness of the soil was larger than the sensing sphere and the sensing geometrical element of the sensor(s) and the open-ended coaxial probe. For each dielectric and capacitance measurement, the container volume and the mass of the soil were recorded, to be used in the computation of the sample’s volumetric water content. The PVC containers were chosen to minimise electromagnetic interference. Prior to the dielectric measurements, the probe was calibrated against air, a shorting block and deionised water. Thereafter, at least three measurements were taken for a given sample, on different parts of the sample’s surface area to ensure that the dielectric constant measurement was an accurate representation of the entire sample. Contact between the probe and the soil was carefully maintained to ensure that there was no air trapped between them. Figure 2 depicts a typical instrument and sample setup used in the experiment.

The dielectric probe measurements were followed by measurements using the four capacitive sensors (explained in detail in the next section). Lastly, a sub-sample was retrieved for subsequent gravimetric and volumetric water content, θ, calculations, using the soil sample dry density derived from the known volume of the container and the measured soil mass [79]. It is worth mentioning that the sub-samples for oven drying were retrieved from the uppermost layer of the sample (around 10 mm) which was estimated to be within the sensing volume of the various probes used. This was to ensure that the sensor outputs were calibrated against a representative volume. The approach undertaken to conduct the capacitive measurements is described in the next section. 

### 4.2. Capacitive Sensor Measurements

Once the sample was prepared, the following measurement protocol was followed for each of the sensors. Firstly, a measurement was conducted whilst the sensor was free in the air and recorded as an air measurement. Subsequent measurements were undertaken by placing the sensor against the surface of the sample and by applying a weight (minimum 200 g) on top of the sensors to ensure a good soil-sensor contact was maintained, without any noticeable air gap between the soil and the sensor. Air gaps could potentially lead to errors in the capacitive reading and thus in the soil water content estimation. To ensure the soil-sensor contact was maintained, a slightly heavier load was used for sensors with a larger footprint. Once the full contact was maintained and consistent readings were obtained from the sensor, the data was recorded on a computer. This procedure was repeated typically three times for each sample to obtain readings that were accurate representations of the entire sample. Once the air and sample readings were recorded, the air reading was subtracted from the reading taken from the sample. This was done to minimise the effect of the environment (such as humidity and temperature) on the measurements. Moreover, this can be considered as a basic and simple calibration to normalise the measurements with respect to the air reading.

It is worthwhile to note that the effect of pressure on the output of the sensor was tested through a separate set of experiments. The sensor readings were monitored while various pressures were applied to the sensor as it was sitting on a flat surface. Once full contact was maintained between the sensor and the material under test, changing the amount of pressure was proven to have no impact on the readings (results are not shown). 

The approach for the Rectangular sensor was slightly different due to its multiple-electrode design. As this sensor is comprised of twelve sensing electrodes, twelve readings were obtained from a ‘single’ measurement. In addition, due to the use of 12 electrodes and the relatively larger size of the sensor unit compared to the Circular sensor, some variations were observed in the readings (results are shown later in the paper in Section 5.2 and Section 5.7). This is likely due to the fact that despite the sample surface being levelled, there still existed some relative surface roughness which may have created an uneven contact between the soil and some of the electrodes, causing noticeable dissimilarities between different electrode readings. To overcome this issue, instead of averaging the twelve readings, which was the approach adopted in the work previously reported by Orangi, et al. [80], the maximum reading among the twelve readings for a given measurement was used for the analysis. Based on the observations throughout this work and the findings highlighted by Orangi, Withers, Langley and Narsilio [80], it was assumed that for a given sample, the larger reading values were derived from the better soil-sensor contact conditions and thus more representative of the true soil water condition. Further details are included in the following section. 

With regard to the PCB sensor, the measurements were conducted without using a weight; the sensor was simply held by hand against the sample surface whilst ensuring full contact was maintained. The measurements with the Circular and AOGAN sensors were conducted as described in the general procedure.

## 5. Results, Analyses and Discussion 

Firstly, the results are summarised for each sensor and soil sample and individual calibrations are derived. Next, an evaluation of the applicability of a single calibration (all soil types) for each sensor is presented. This is followed by an evaluation of the efficacy of using a separate calibration for sandy soils (i.e., combining the Brighton Group Sand and Silty Sand data—referred to as sand group in this manuscript) and for cohesive soils (i.e., combining Camb Clay and Bun Silt data—referred to as clay groups in the manuscript) for each sensor. The dielectric properties of the samples are then estimated based on capacitive measurements and lastly, the effect of soil sensor coupling and surface roughness on sensor performance is evaluated by using the results of the present study and of Orangi, Withers, Langley and Narsilio [80].

### 5.1. Circular Sensor

**Figure 3:**
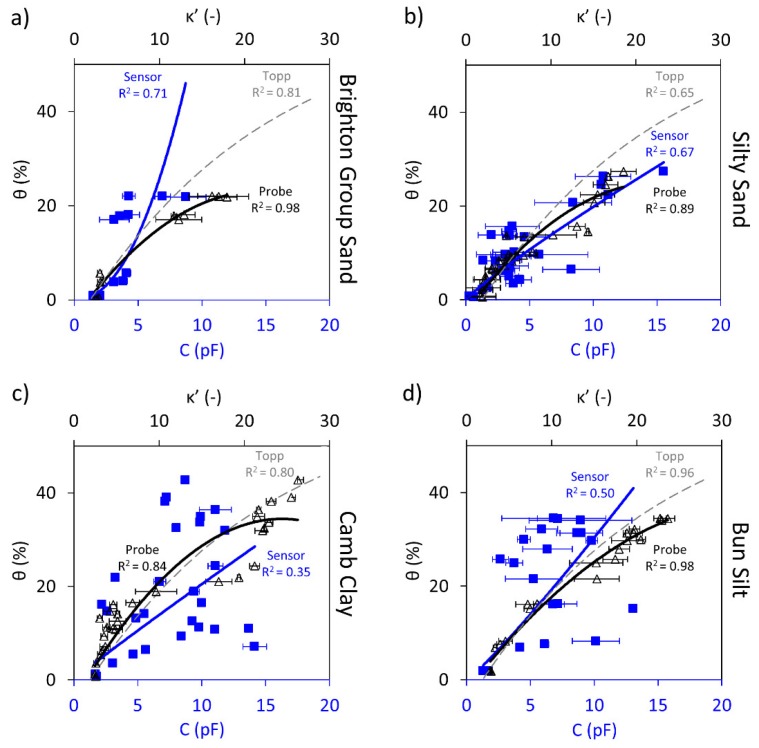
Circular sensor capacitance, C, readings (blue square markers and trendline) and dielectric constant, κ’, measurements (black triangular markers and trendline) shown against the volumetric water content, θ; the Topp equation is also shown (dashed grey trendline). Shown for: (**a**) Brighton Group Sand (**b**) Silty Sand (**c**) Camb Clay and (**d**) Bun Silt.

Results of the Circular sensor performance against volumetric water content are shown in Figure 3. Each plot includes capacitance readings measured by the sensor and the dielectric constants from the dielectric probe versus the volumetric water content for each soil sample. The capacitance and dielectric measurement data are plotted with blue squares and black triangular markers, respectively. Moreover, the “expected” volumetric water content based on the Topp calibration and the measured dielectric constant (Equation (4)) is superimposed on the plot (dashed grey trend line).

Figure 3 illustrates an increasing trend captured by the Circular sensor for the four samples; however, with different levels of accuracy. As was explained in the theory and background section, an increase in the soil volumetric water content causes an increase in the dielectric constant of the soil, due to the larger number of water dipoles. An increase in the capacitance is also expected since Equation (4) shows that the relationship between capacitance and the dielectric constant is directly proportional. 

For the Brighton Group Sand and the Silty Sand samples (i.e., the coarse-grained soil samples), the sensor was able to capture the variation of water content with capacitance; however, the correlations show errors in the order of 10%. 

For the Camb Clay and Bun Silt (i.e., the fine-grained soil samples), it is observed that the Circular sensor is able to capture the increasing trend; however, the correlations compared to the sand samples are significantly less obvious, as shown by the significantly reduced coefficient of correlation, R^2^. 

The Topp calibration predictions shown by the dashed grey trendlines show a good agreement up to approximately 5% and 12% for the Brighton Group Sand and Silty Sand, respectively. However, as the water content increases, the Topp calibration overestimates the data. Surprisingly, the Topp calibration seems to fit the measured data for the cohesive soils tested here better than for the sandy soils, which is contrary to the assumptions made in deriving the calibration. It is worth mentioning that the R^2^ for the Topp calibration describes how well this calibration captures the data. 

Table 2 quantitatively summarises the best fit models and the measure of errors obtained for the Circular Sensor and the dielectric probe for the four soil samples. Additionally, the universal Topp calibration performance for each soil type is assessed. The results in the table confirm that the Circular Sensor can capture the increasing trend for the sand samples. Furthermore, for the cohesive samples, a very weak increasing trend could be identified from the data for both Camb and Bun samples, however, with an unacceptable level of accuracy and large errors. Despite the relatively low R^2^ values for capacitive readings, it is worth mentioning that the dielectric probe measurements have shown some similar variations compared to the corresponding capacitive measurements for the first two soil samples.

A two-fold cross-validation analysis for the Circular Sensor was conducted (similar to other sensors in the next sections). In this analysis, half of the data is used for conducting a calibration and subsequently, the remainder of the data is used for validation. The results are summarised in Table 3 which further proves the weak performance of the Circular Sensor based on the low R^2^ values and large errors.

It is important to note that during measurement with this sensor, an instability issue of the reading occurred and the sensor could not retrieve any readings for some samples. This instability issue is attributed to the controller board of the Circular Sensor, as well as the lack of permanent insulating coating on the electrodes which may have created short circuits during measurements and the difficulties in achieving full soil-sensor coupling.

These reasons collectively have resulted in the relatively low performance of the Circular Sensor, particularly for fine-grained soils. Despite its weak performance, Circular Sensor demonstrates promising potential in detecting the variation in soil water content using non-invasive capacitive sensors. In view of the observed limitations, we have developed the Rectangular Sensor, whose results are described next.

### 5.2. Rectangular Sensor

The Rectangular sensor comprised 12 electrodes and each electrode returned a reading upon being in contact with the soil sample. Essentially, since the water content of the sample is envisaged to be homogeneous due to the sample preparation method, it is expected that the output from the 12 electrodes are similar or only with marginal variations due to electrodes layout. Therefore, it would be reasonable to report the mean of the 12 readings as the capacitive value for a given sample. This approach was adopted in a previous study by Orangi, Withers, Langley and Narsilio [80]; however, the Rectangular sensor performance was deemed unsatisfactory in that preceding study. In this work, the maximum reading (instead of the average) was adopted. The rationale for this choice was explained previously in Section 4.2 and the results are given in Section 5.7. 

**Figure 4:**
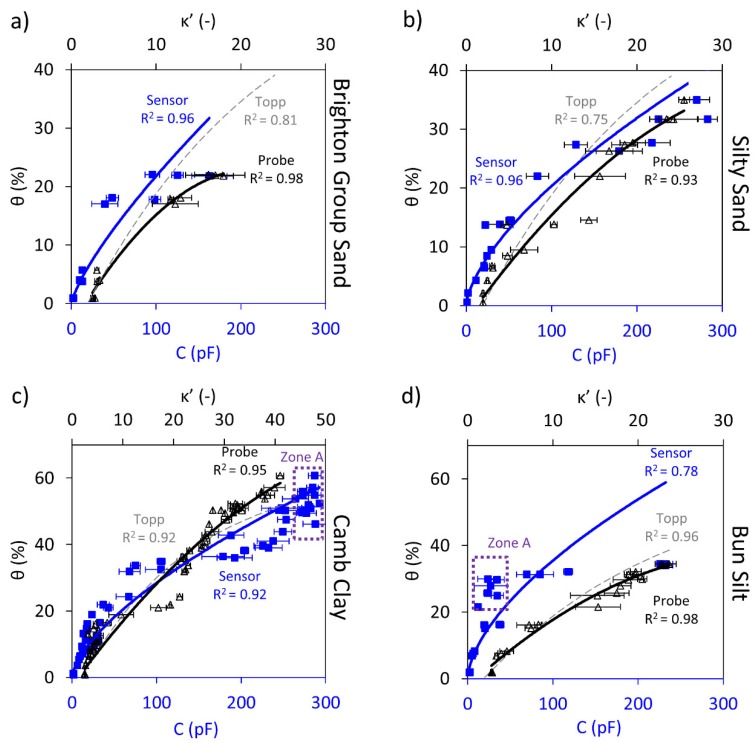
Rectangular sensor capacitance, C, readings (blue square markers and trendline) and dielectric constant, κ’, measurements (black triangular markers and trendline) shown against the volumetric water content, θ; the Topp equation is also shown (dashed grey trendline). Shown for: (**a**) Brighton Group Sand (**b**) Silty Sand (**c**) Camb Clay and (**d**) Bun Silt.

Figure 4 summaries the results of the Rectangular sensor against the volumetric water content. With the Brighton Group Sand and the Silty Sand samples, the (directly proportional) trend between the sensor readings and the volumetric water content can be clearly seen. In Figure 4a,b (coarse-grained soils), the sensor readings (blue square markers) show a strong correlation with increasing volumetric water content, which resembles the variations observed in the measured real dielectric constants (black triangular markers).

For the fine-grained soils tested, the proportional trend between the capacitive reading and the volumetric water content can be clearly seen for both samples, as opposed to for the Circular sensor, where this trend had a weak resemblance due to the discussed limitations. Moreover, the instability of readings and the limited measurement range issues encountered by the Circular sensor are resolved here. The improvements are considered to be the result of the new controller as well as the larger geometry of the sensor, which facilitated maintaining full contact between the soil and sensor. However, it can be seen that electrodes returned almost constant readings despite the increase in sample water content (Zone A in Figure 4d), presumably due to the partial contact of electrodes for the Bun Silt samples with rougher soil surfaces. Indeed, soil trimming and surface smoothing were more difficult to achieve for the Bun Silt samples with water content between 20% and 30% (close to optimal water content). Otherwise, the trend is well captured by the Rectangular sensor for each of the sandy and cohesive samples.

The issue with the limited measurement range appeared for this sensor as well; however, at a much higher water content than when using the Circular sensor. As a result, the measured capacitive data beyond approximately 45% volumetric water content form a cluster of data points, as illustrated in Zone A of Figure 4c. That is, beyond approximately 40% water content, the sensor reached its upper limit and could no longer capture variation in the water content. Although the sensor was unable to differentiate the water content beyond this threshold, this situation is not commonly encountered in practice, since the threshold would be generally above the soil field capacity for most of the soils. Hence, this is not considered a major issue for the Rectangular sensor. 

Table 4 summarises the correlations obtained for the Rectangular sensor and the dielectric probe against volumetric water content. A power fit between the capacitive reading and the volumetric water content describes the correlations and indicates a good agreement (refer to Figure 4). 

Based on these results, the Rectangular sensor appears to have effectively predicted the variation of the volumetric water content. However, the size of the sensor may hinder good soil-sensor coupling (e.g., Bun Silt). This is thought to be due to the relatively larger size of the Rectangular sensor which made working with samples such as Bun Silt harder where the sample surface presented large undulations.

The results of the cross-validation study for the Rectangular sensor are summarised in Table 5.

The calibration function for each soil, which was based on half of the experimental data, is shown to be able to predict the behaviour of the remainder of the dataset with a strong correlation. Moreover, the RMSEs of the validation dataset are comparable to the ones from the calibration. This analysis further demonstrates the capability of the Rectangular sensor in predicting the variation in soil moisture content of different soil types. Nonetheless, there are limitations with regard to the multiple electrode design as well as the size of the sensor; these limitations were addressed in the development of the fourth sensor. 

### 5.3. PCB Sensor

**Figure 5:**
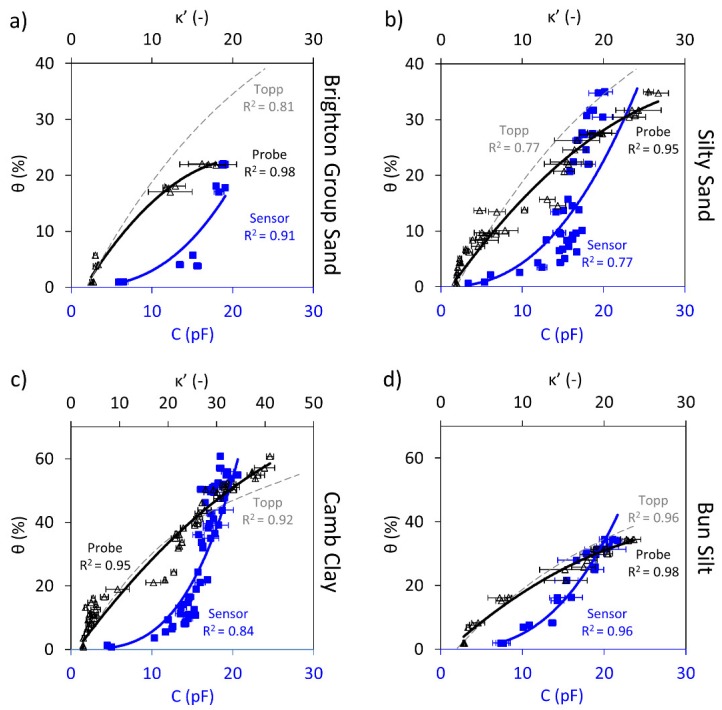
PCB sensor capacitance, C, readings (blue square markers and trendline) and dielectric constant, κ’, measurements (black triangular markers and trendline) shown against the volumetric water content, θ; the Topp equation is also shown (dashed grey trendline). Shown for: (**a**) Brighton Group Sand (**b**) Silty Sand (**c**) Camb Clay and (**d**) Bun Silt.

The PCB sensor had a large geometry and sensing area compared to the Circular sensor, however, was smaller than the Rectangular sensor. Therefore, maintaining good coupling between the soil and the PCB sensor was relatively easy due to its size and being a single electrode. Figure 5 depicts the variation of PCB sensor outputs with water content, alongside the measured dielectric constants for all of the samples. A clear trend between capacitive readings and volumetric water content is shown across the samples; however, with a much lower sensitivity to water content beyond 15%. For the soil samples shown in Figure 5, the change in sensor readings is significantly larger for a water content variation from dry to approximately 15%, than for a water content variation beyond 15%. This clearly indicates that the PCB sensor is able to distinguish the changes in the water content; however, with a significantly reduced ability to accurately estimate the water content beyond 15%. This further highlights the limited capability of this sensor to estimate the water content of the fine-grained soils tested here, which can generally have water content above 15% in natural conditions.

Table 6 summarises the calibration obtained for the PCB sensor as well as for the dielectric probe. The values show that the performance of the PCB sensor in predicting the water content is comparable to that of the dielectric probe; nonetheless, with the aforementioned limitation regarding the measurements for samples with water content beyond 15%, indicated by higher errors despite high R^2^ values (see Table 6).

Cross-validation analysis was performed for the PCB sensor and the results are summarised in Table 7. The results suggest that the PCB sensor is able to capture the increasing trend between the capacitive reading and volumetric water; however, the errors are relatively large. The large errors are assumed to be the result of the reduced sensitivity of the PCB sensor.

From these results, it is concluded that the geometry of the PCB sensor rectifies the sensor-soil contact issue. Moreover, the design is further enhanced by being a single electrode (similar to the Circular sensor) as opposed to being a multiple electrode design (e.g., the Rectangular sensor). However, there is the issue of sensitivity, caused by the controller board of the PCB sensor, which precludes accurate and reliable estimation for soils with water content above 15%.

### 5.4. AOGAN Sensor—An Integrated Sensor Designed Utilising the Advantages of the Previous Sensors

It is shown in the previous sections that the Circular, Rectangular and PCB sensors are able to capture changes in soil water content. However, there are advantages and limitations associated with each of the sensors.

In summary, the advantages are as follows: Firstly, a single electrode helped to maintain superior soil-sensor contact (Circular and PCB sensors) and showed a lower sensitivity to high frequency surface undulations relative to the sensor size (PCB sensor). Secondly, the controller board of the Rectangular sensor provided a reliable capacitive sensing range, facilitated stable readings and enabled working with samples with high water content. Moreover, the insulating agent coating the Rectangular sensor effectively prevented potential short circuit issues when dealing with very wet soil samples. Similar to that of the PCB sensor, the larger geometry of the Rectangular sensor created a better platform for conducting the measurements and maintaining good soil-sensor coupling or contact. Nonetheless, the significantly larger design of the Rectangular sensor proved to be problematic for some measurements (e.g., Bun Silt samples). Furthermore, from the perspective of conducting the measurements, the shape of the Circular sensor proved to be superior than that of the other two sensors, in maintaining contact and in the ease of use. 

On the other hand, key limitations include the inadequate sensing range and the instability issue of the Circular sensor, the multiple electrodes and significantly larger geometry of the Rectangular sensor and the limited sensitivity issue of the PCB sensor. 

The AOGAN sensor was designed by combining the identified advantages of the Circular, Rectangular and PCB sensors and eliminating their identified limitations. As such, the design of the AOGAN sensor was inspired by the shape and single electrode design of the Circular sensor and the larger geometry of the PCB sensor and incorporated a board designed and printed to act as the sensing component. This sensing component was almost three times larger than that of the Circular sensor to help with increasing the sensing range and was accompanied by a waterproof agent (similar to the Rectangular and the PCB sensors) to eliminate the potential short circuit issue. It is important to note that using an insulating film creates a sensor that is measuring two capacitors in series: formed by the insulating film and the soil, respectively. However, since the thickness of the insulating film was less than 0.05 mm, we assumed that the effect on the soil water content estimation was minimal. The larger geometry and the single electrode design improved practicality for conducting measurements. Furthermore, the sensing component was controlled by a board similar to the one used in the Rectangular sensor (which provided a more stable and larger sensing range and readings). The sensing component and the controller board were then connected to an Arduino-based board which communicated with a laptop. The program used for controlling the AOGAN sensor was the same as the one used for the Rectangular sensor. 

**Figure 6:**
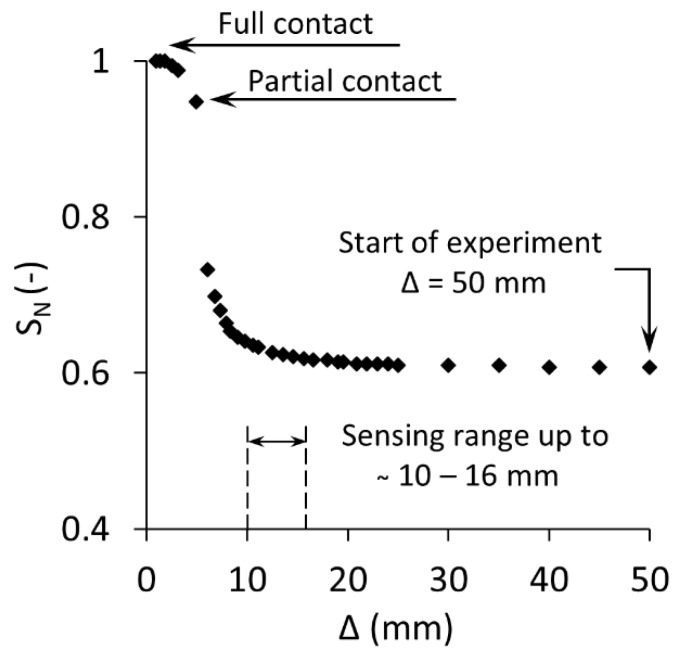
Normalised Sensor reading, S_N_, against the distance between the soil sample and sensor Δ.

An experiment was designed to estimate the sensing range of the sensor. Based on the methodology described by Orangi and Narsilio [71], a wet soil sample was prepared with the Silty Sand and was placed on a lab jack. The initial distance between the sample and the sensor was at 50 mm and the sample has subsequently approached the sensor at small increments controlled by a dial gauge. The capacitance measurements were recorded until a full contact between the soil and sensor was achieved. The result of this experiment is shown in Figure 6. The normalised sensor output, S_N_, is plotted against the separation between the soil sample and the sensor, Δ. The figure shows that the sensing range is within 10 to 16 mm. We have estimated, therefore, that the depth that the sensor is able to estimate soil water content is around 10 mm. It is worth mentioning that the full contact between the soil and the sensor resulted in a more reliable sensor output.

**Figure 7:**
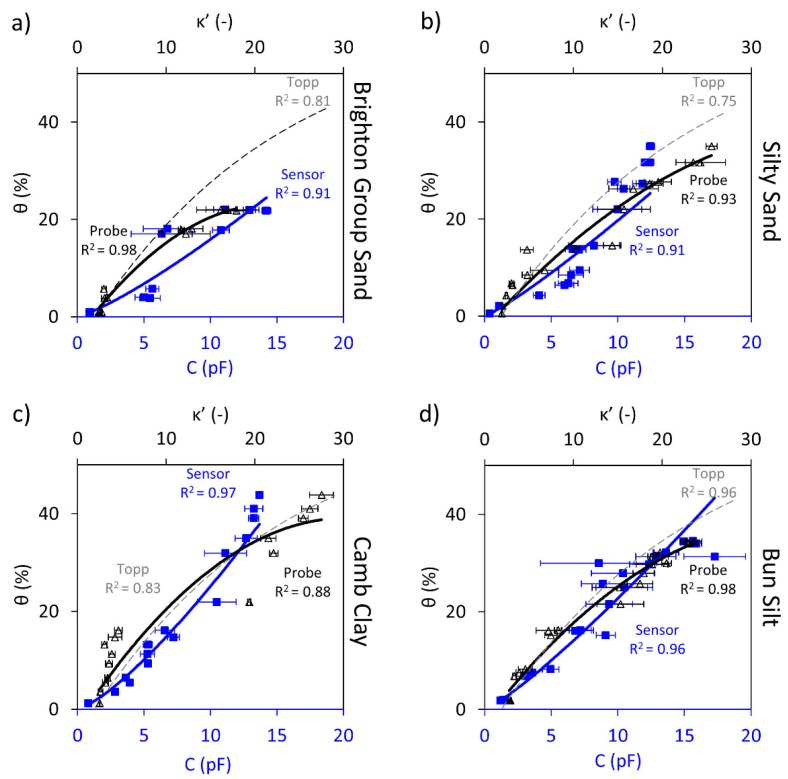
AOGAN sensor capacitance, C, readings (blue square markers and trendline) and dielectric constant, κ’, measurements (black triangular markers and trendline) shown against the volumetric water content, θ; the Topp equation is also shown (dashed grey trendline). Shown for: (**a**) Brighton Group Sand (**b**) Silty Sand (**c**) Camb Clay and (**d**) Bun Silt.

Figure 7 depicts the strong correlations obtained between the AOGAN sensor capacitive readings and water contents for all of the samples that were tested. Minimal issues were encountered with regard to soil-sensor coupling, instability, sensing ranges and low sensitivity to water content variation, which were observed for the previous sensors. It is assumed that this enhanced performance is a result of the advantageous features incorporated in the design of the AOGAN sensor.

It is important to note that the preparation of samples plays a key role in for the surface quality status of the soil samples, which impacts the quality of the soil-sensor contact or coupling. In practical applications, it is crucial to note that the deployment of sensors in the field requires the development of a mechanism that maintains full soil-sensor contact. This is to ensure a reliable sensor performance, as was observed during the laboratory measurements where the contact was maintained by using a weight. 

As shown in Figure 7d, there are some samples for which the standard deviations of the measurements (error bars) are relatively large, possibly due to the contact issue between the soil and the sensor. These are some of the same samples for which the rectangular sensor was unable to capture the soil water content variations.; however, the AOGAN sensor showed less sensitivity to surface undulations due to its smaller size. The large standard deviations may, therefore, be the result of inadequate trimming of the samples and not the sensor hardware. Additionally, the inconsistency in the soil water content of a given sample was most likely not the reason for such discrepancies, since the samples were mixed thoroughly and cured during the preparation stage. As a result, it can be assumed that the water content was relatively homogenous for a given sample. The errors involved in soil water content estimation based on the calibration for this sensor were less than 5%. A summary of the results is shown in Table 8.

Overall, considering the improved performance attributes, the AOGAN sensor shows great potential to estimate the soil water content non-invasively. 

The cross-validation analysis results for the AOGAN sensor are given in Table 9. Overall, the statistical measures for the calibration and validation functions show the strong capability of the AOGAN sensor to capture the variations of the volumetric water content of soils. The values in the table also show the superior performance of this sensor compared to the other three sensors (See Table 3, Table 5 and Table 7). 

### 5.5. Effect of Soil Type on the Calibration of the Sensor

It is reported in the literature that the relationship between a soil’s electrical properties and its water content is determined by the soil type [81,82,83]. It is, therefore, imperative to evaluate the extent of soil type effects on the performance of the sensors in this work. In the results section, we showed that for sensors with good performance, a separate calibration could adequately describe the data for each soil sample. However, employing a single calibration for each individual soil type may not be practical and could become a tedious task in practice. This likely explains why a single calibration has been adopted for a range of soil types in previous studies (e.g., see [53,54,84,85]). The empirical Topp calibration proposed by Topp, Davis and Annan [53] was based on soils ranging from heavy clay to sandy loam; however, it is unable to accurately estimate the water content of some soil samples tested in the current study and also in a number of previous studies (e.g., [86,87,88,89,90]). Nonetheless, it is currently one of the most widely used empirical calibrations for estimating water content using soil dielectric properties. Therefore, following the same approach, we evaluated the efficacy of using a single calibration for each sensor in the present study. Data from the four samples was collated as a dataset and the performance of each sensor and the dielectric probe was analysed against it. 

**Figure 8:**
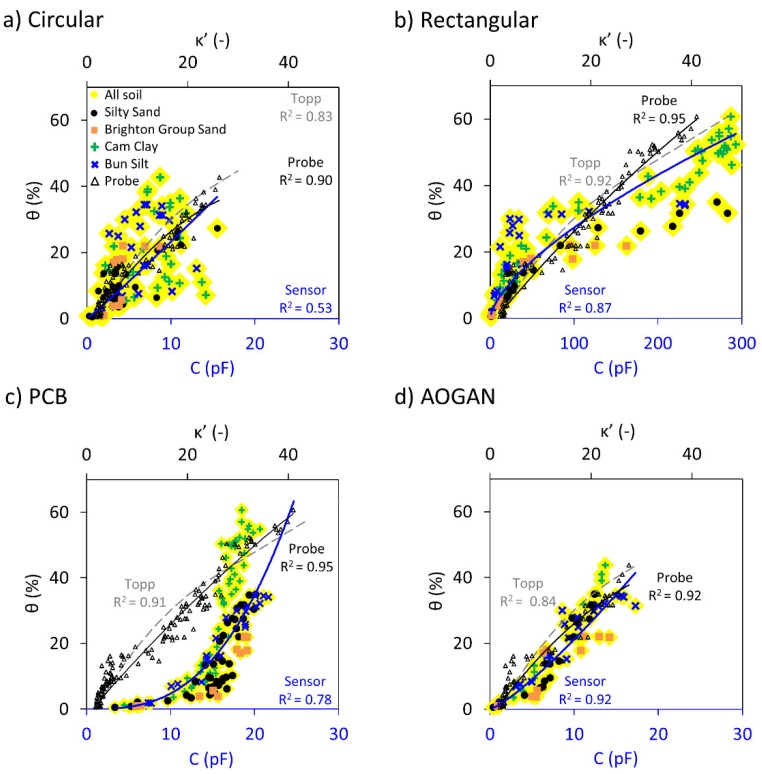
Combined capacitance, C and dielectric constant, κ’, data versus volumetric water content, θ, for: (**a**) Circular, (**b**) Rectangular, (**c**) PCB and (**d**) AOGAN sensors. Highlighted yellow markers capture the capacitive data for all soils and different markers represent different soil samples captured by the blue trendline. Black triangular markers and dashed trendlines correspond to dielectric measurements. Topp calibration is shown by the dashed grey trendlines.

Figure 8 shows the collated capacitance readings and real dielectric constant data for the four samples plotted against volumetric water content. The Topp calibration is also shown for comparison. For clarity, the standard deviations of the measurements have been removed. The collation of capacitive data is shown by highlighted yellow markers and different markers correspond to different soil types, captured by a blue trendline. The capacitive readings are fitted with a power function as in the previous section. The measured dielectric constants and the corresponding trendlines are shown by black triangular markers and black trendlines, respectively. 

As seen previously, the Circular sensor was unable to capture the variation of soil water content for the cohesive samples (Figure 8a); it was better able to capture the trend for the sandy samples. In addition, the instability issue occurred for all of the soil samples. Therefore, due to these two issues, combining the data for this sensor to evaluate the efficacy of a single calibration has resulted in a weak correlation (R^2^ = 0.53) and large errors (≈10%) as presented in Table 10.

By contrast, for the Rectangular sensor shown in Figure 8b, a single calibration describes the dataset with relatively good agreement and resembles the calibration obtained using the dielectric constant data. Nonetheless, using the single calibration for the sensor may clearly lead to overestimating the water content of the sand samples (i.e., Brighton Group Sand and Silty Sand samples that are shown by orange and black markers, respectively) for soils with moisture content above approximately 20%. This is highlighted in Table 10 showing a lower R^2^ value (R^2^ = 0.87) and relatively large errors (≈7.5%) compared to the calibration for individual soil samples (Table 4). Considering the capacitive measurements obtained by this sensor, one can see two distinct clusters of data versus volumetric water content. These two clusters, in fact, can be categorised as sandy soils and cohesive soils. Thus, two separate calibrations are expected to better estimate the water content for each cluster. 

With regard to the PCB sensor, it can be seen in Table 10 that employing a single calibration for describing the capacitive reading versus volumetric water content has resulted in a lower R^2^ than for the Rectangular sensor and larger errors. This further suggests that deriving separate calibrations for sand and clay groups can help in better describing the water content variation using the capacitive reading. However, the performance of the PCB sensor was proved to be questionable in the previous sections.

For the AOGAN sensor depicted in Figure 8d, a single calibration is shown to effectively capture the capacitive readings and volumetric water content relationship for the combined dataset (see Table 10). Compared to the previous sensors, due to a weaker contrast between the measurements made for the sand and clay groups, it is suggested that there is a lesser dependency on the soil type for the AOGAN sensor. The improved geometry and controller board design of the AOGAN sensor appears to provide better soil-sensor coupling and readings, which are, in turn, less affected by the soil type. Nonetheless, the data is treated separately in two groups in order to refine the correlations. 

Table 10 includes a summary of the data corresponding to the sensors and the dielectric probe calibrations. These calibrations were derived and assessed against the corresponding dataset used for each sensor. It is worth mentioning that the relationship between volumetric water content and dielectric constant data could be adequately described by adopting single calibrations, which are superior to the Universal Topp calibration, as these here become site specific calibrations. 

It is shown that the performance of the AOGAN sensor against the combined dataset is superior to that of the other sensors and that it is less affected by the soil type. However, it is suggested that adopting separate calibrations could provide improved predictions for the sensor. Moreover, for the other sensors, distinct behaviour was observed for the sand and clay groups and relatively larger errors were introduced by adopting a single calibration. 

**Figure 9:**
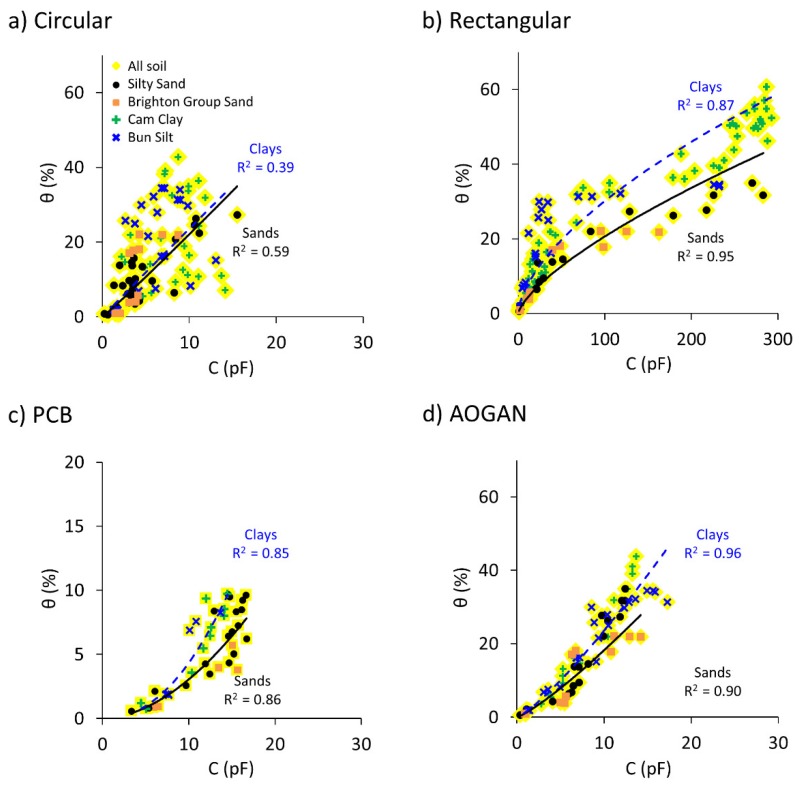
Combined capacitance, C and dielectric constant, κ’, data versus volumetric water content, θ, for the: (**a**) Circular, (**b**) Rectangular, (**c**) PCB and (**d**) AOGAN sensors. Highlighted yellow markers capture the capacitive data for all soils and different markers represent different soil samples. Separate calibrations are used for describing the sand group (black solid trendline) and clay group (blue dashed trendline).

Figure 9 shows for each sensor the combined capacitive readings for all of the soils but data are grouped into sands (described by the black solid trendline) and clays (described by the blue dashed trendlines). 

It was concluded from previous analyses in Section 5.1 and Section 5.5 that the Circular sensor is unable to detect changes in water content of the clayey samples and therefore that the performance of this sensor is not satisfactory at least for the clayey samples. It is unsurprising, therefore, that for this sensor, better performance is observed when using separate calibrations. For the Rectangular sensor, separate calibrations seem to capture the individual groups, rendering smaller errors for each group and higher R^2^ values for the sand group, as shown in Figure 9b and Table 11. For the PCB sensor, the predictive performance has smaller errors for both the sand and clay groups compared with the single calibration; whereas, the performance of the AOGAN sensor is less dependent on the soil type, as shown by only a marginal improvement upon employing separate calibrations. 

It is shown that by employing separate calibrations, all of the sensors obtain better goodness-of-fit and lower error in soil water content estimation. Nonetheless, the predictive performance of the AOGAN sensor improved the least by adopting this approach, which again highlights the superiority of the AOGAN sensor among the other sensors. 

### 5.6. Dielectric Constant Approximation through Capacitive Measurements

It was shown in the previous sections that the readings from the four capacitive sensors correlate (with different goodness-of-fits; 0.57 for the Circular sensor, 0.91 for the Rectangular sensor, 0.87 for the PCB sensor and 0.94 for the AOGAN sensor) with variation in the volumetric water content. On the other hand, it is known that the capacitance of a capacitor is derived by its geometry and the dielectric constant of its material (refer to Equation (4)). Therefore, can the dielectric constant of the material under test be reliably estimated using the capacitive sensor readings? In this section, correlations between the sensor readings and the dielectric constant data (measured by the Agilent dielectric slim form probe) are obtained, for both the sand and clay groups, for each sensor.

**Figure 10:**
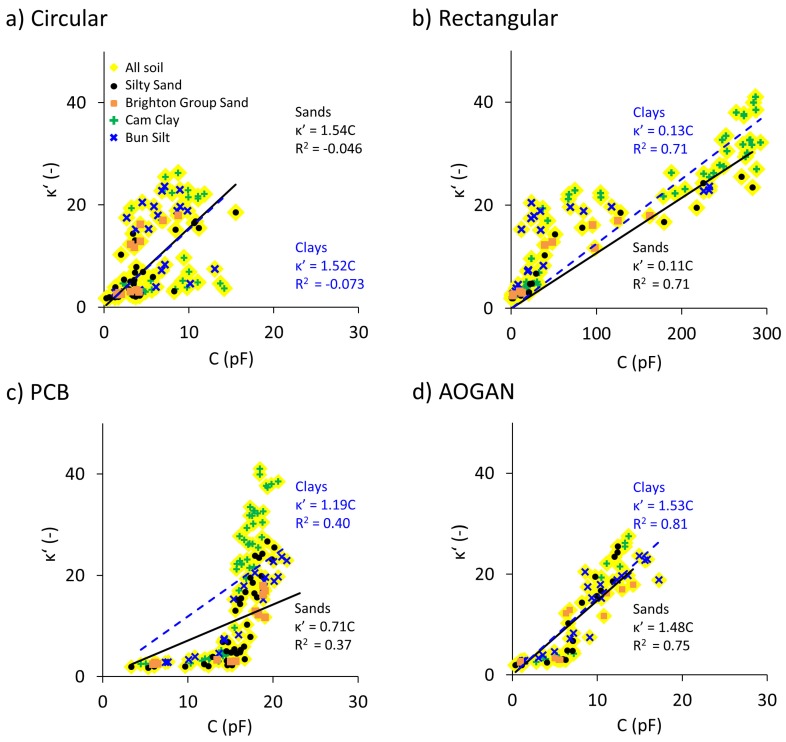
Measured dielectric constant, κ’, data against capacitive, C, readings for the sand (black solid trendline) and clay (blue dashed trendline) groups, for the: (**a**) Circular, (**b**) Rectangular, (**c**) PCB and (**d**) AOGAN sensors.

Figure 10 shows the sensor readings versus the dielectric constant data. For each sensor, the data are divided into sand and clay groups and for each a linear correlation which passes through the origin illustrates the relationship shown in Equation (4). This approach enables estimation of the geometry factor, g, for the sensors and the dielectric constant from the sensor output. 

The performance of the Circular sensor was shown to be unsatisfactory in the previous section and this is verified by the relationship obtained here, represented by the trendline. The Rectangular sensor shows stronger correlations for both soil groups. It is shown that the equations describing the relationships for the sand and clay groups are quite similar, which, in turn, result in similar geometry factors. The geometry factor for any capacitor is a constant value and this is somewhat explained by the similar slopes of the two trendlines of two soil groups. For the PCB sensor, as was explained previously, due to the weak signal-to-noise ratio (i.e., lower sensitivity), the correlation between the sensor reading and the measured dielectric constant is not strong for any of the soil groups. With regard to the AOGAN sensor, due to its improved design and performance, correlations between the sensor readings and the measured dielectric constant are strongest. In addition, the correlation between capacitive readings obtained from the AOGAN sensor and dielectric constant data are similar for both soil groups and return almost identical geometry factors (See Figure 10d). 

The superior performance of the AOGAN sensor (followed by the Rectangular sensor) is demonstrated in this analysis by the strongest proportionality between the sensor readings and the measured dielectric constants. 

In the next section, the effect of sample preparation on the performance of the sensors is discussed.

### 5.7. Effect of Surface Contact and Roughness

Contact between soil and a sensor and the roughness of a sample’s surface, are demonstrated by the experimental data to be important factors influencing the quality of measurements made with the sensors. It was suggested by Orangi, Withers, Langley and Narsilio [80] that the soil-sensor coupling and soil surface roughness could be responsible for the poor performance of the Rectangular and PCB sensors in their work. Hence, in this section, the raw data from Orangi, Withers, Langley and Narsilio [80] are re-examined to investigate the importance of these factors.

**Figure 11:**
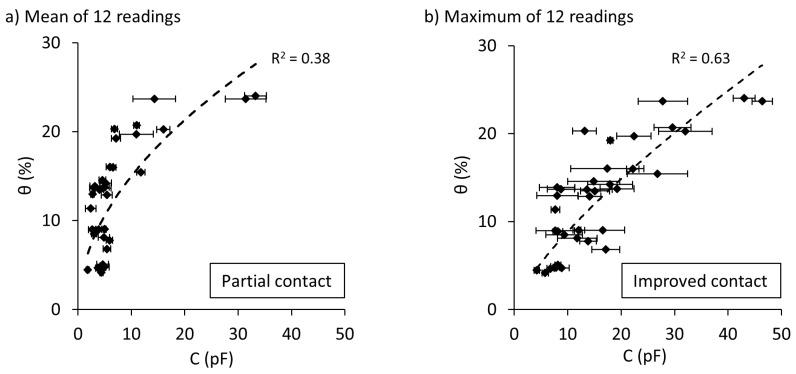
Effects of soil sensor coupling on the estimation of volumetric water content, θ, using the Rectangular sensor on the Brighton Group Sand: (**a**) Mean of 12 readings (Partial contact), (**b**) Maximum of 12 readings (Improved contact). Data adapted from [80].

In this present work, of the twelve readings obtained from a single measurement with the Rectangular sensor, the maximum reading was taken as the representative capacitance value instead of using the mean value. This decision was based on the result of the following analysis conducted on the experimental data published by Orangi, Withers, Langley and Narsilio [80] on the Brighton Group Sand. That is, for a set of experiments conducted on the Brighton Group Sand, we have adopted two methods for the analysis. Firstly, for each sample, the variation of capacitance with water content was investigated, using the mean value of the readings from 12 electrodes. The second approach was to use the maximum of the 12 readings obtained by the Rectangular sensor. The results are shown in Figure 11.

As illustrated in Figure 11, the ability of the Rectangular sensor to detect water content variation is improved when the maximum value of the 12 readings is considered as the capacitance value (Figure 11b). It is observed that the R^2^ value increases by more than 35% when choosing the maximum sensor reading instead of the mean (Figure 11a). Nonetheless, for the Rectangular sensor and the Brighton Group Sand, the value of R^2^ obtained in the above study (R^2^ = 0.63) is less than the value obtained in the current study (R^2^ = 0.95, Figure 4a, Table 4). This is likely attributed to partial contacts exist during the previous experimentation. Therefore, these results suggest that soil-sensor coupling can significantly affect the performance of the sensors. The issue associated with the number of electrodes discussed in Section 4.2 does not apply to the PCB sensor or other sensors comprising a single electrode; however, by using the data for the Rectangular sensor we can demonstrate the importance of effective coupling, as well as justifying the rationale behind utilising the maximum output as the reading of the Rectangular sensor. 

**Figure 12:**
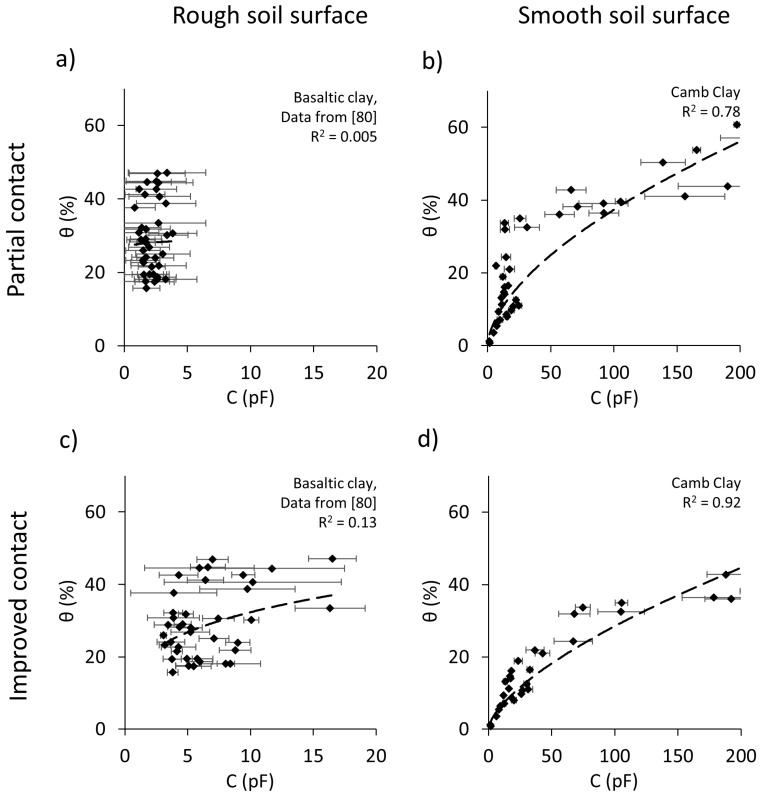
Effect of soil surface roughness and sensor contact on the performance of sensors for two clay samples: Basaltic Clay and Camb Clay samples. Basaltic Clay data are from Orangi, Withers, Langley and Narsilio [80].

The surface of the soil samples tested by Orangi, Withers, Langley and Narsilio [80] were less smooth than those used in the current study. Figure 12 shows the relationship between the Rectangular sensor readings and the water content data for a Basaltic Clay tested by Orangi, Withers, Langley and Narsilio [80] and for the Camberwell Clay tested in this work. Figure 12a shows the capacitive values (recorded as the *mean* of the 12 readings) of the Basaltic Clay samples tested by Orangi, Withers, Langley and Narsilio [80], which had *rough* surfaces. It can be seen in this figure that the correlation between the sensor readings and the water content is very weak. Figure 12b shows the *mean* capacitive readings for the Camb Clay, which had *smooth* surfaces. It can be seen that the correlation between the sensor readings and the water content improves significantly with a smoother soil surface. However, the soil-sensor(s) coupling is considered to not be evenly maintained, due to using the mean of 12 readings. Figure 12c shows data for the Basaltic Clay in which the *maximum* of the 12 readings was taken as the sensor reading. Improved contact and correlation can be seen compared with Figure 12a; however, since the surfaces of the samples were *rough* the correlation is still weak.

The weak performance observed in Figure 12a,c is due to the rough soil surfaces associated with sample preparation. Moreover, the standard deviation of the capacitance values from multiple measurements made for a given sample are larger compared to the results of the current study. This appears to be the result of samples having uneven surfaces, creating large variations in sensors readings. However, in Figure 12d, a significant improvement in the quality of the data is shown, owing to the *smoothness* of the sample surfaces as well as good soil-sensor contact, achieved by taking the *maximum* measured value of the 12 readings. Therefore, by comparing the results of the current study with the previous study [80] for two clay samples using the Rectangular sensors, it can be concluded that the surface roughness of samples and the soil-sensor contact play vital roles in ensuring reliable measurements. 

**Figure 13:**
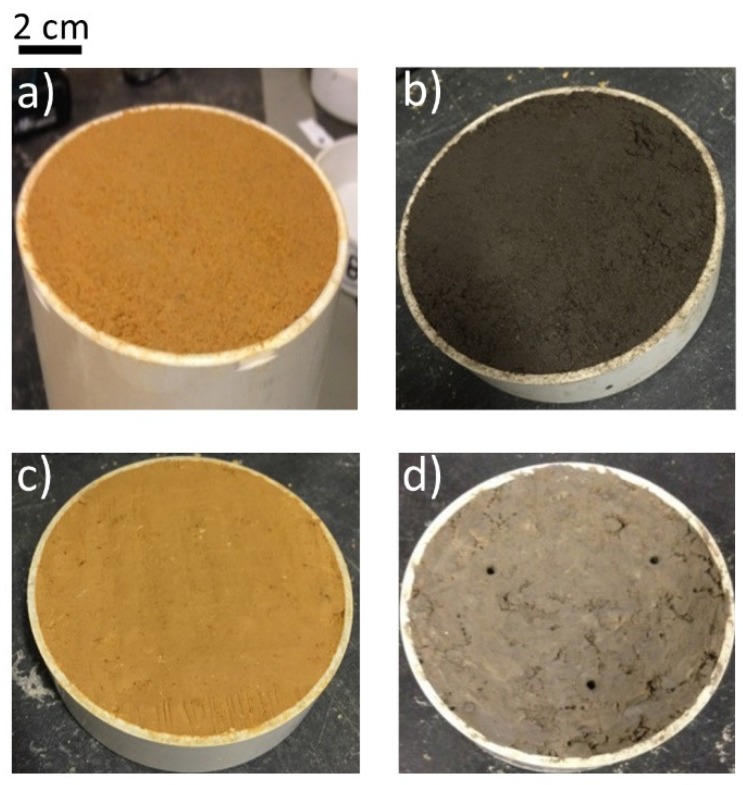
Typical examples of the prepared samples of: (**a**) Brighton Group Sand, (**b**) Silty Sand and (**c**) Camb Clay from the present study and (**d**) Basaltic Clay from Orangi, Withers, Langley and Narsilio [80].

The Camb Clay and Bun Silt samples had similar surface finishes. Moreover, Figure 13d shows an example of the surfaces of clay samples (i.e., Basaltic Clay) used by Orangi, Withers, Langley and Narsilio [80]. The surface roughness visible in this figure was a result of inadequate trimming of the samples during the preparation step by Orangi, Withers, Langley and Narsilio [80]. It is thought to be the underlying reason for the weak performance of the Rectangular and PCB sensors shown in Figure 12, compared to the results of the current study.

Based on the analysis conducted in this section, it is suggested that a smooth surface as well as full coupling between the soil sample surface and the sensor are key factors in ensuring that sensors can effectively estimate the soil water content and detect its variations. 

## 6. Potential Applications and Limitations

The new sensors developed in this work do not require insertion into the soil, nor do they require subsamples to be retrieved for subsequent gravimetric calculations. Furthermore, due to their non-invasive nature and the speed of measurements (milliseconds to retrieve a reading), repetitive measurements at the exact same location are possible, which makes these sensors suitable for large-scale near surface soil water content monitoring. A potential application in the agriculture sector could be high spatial and temporal resolution mapping of surface soil moisture, beneficial for farm management. The non-invasive characteristic of the sensors enables frequent soil moisture measurement across a farm, which aids in decision making concerning sowing time, irrigation and fertiliser scheduling. Moreover, the sensors can provide ground truth data to calibrate satellite image and remotely sensed soil moisture data. Additionally, the new sensors can be used as a real-time monitoring system of near surface soil water content, for quantifying the risk of bushfire and generating warnings to the pertinent authorities when the soil water content falls below a certain threshold. Another potential application includes the use of the new sensors as a quick way of estimating the moisture content of sub-base and stockpile materials in the road construction industry. However, regardless of the application, the quality of the surface where the measurement is conducted against is an imperative parameter. Therefore, a smooth surface must be somehow achieved in the agricultural fields. With the current sensor design, however, it may be impractical to be used as a field sensor. Thus, it is deemed necessary that further mechanical features are added to the sensor to help with soil surface preparation in the field. On the other hand, in road construction applications, when quality assurance (i.e., water content and density measurements) is conducted, the surface of the sub-base layer after each run is considered to be adequately flat and smooth for direct measurement with this sensor. 

Regarding limitations of the sensors, it is important to state that the estimation of soil water content is currently limited to approximately 1 cm deep, based on the width of the electrodes, W and their spacing, S, estimated using the approach proposed by Gao, Zhu, Liu, Qian, Cao and Ni [49]. Thus, measurements of deep soil moisture (e.g., up to approximately 1 m), which may be required in precision agriculture, particularly for the horticulture sector, cannot be conducted directly by the sensor from the surface. However, there are crops with shallower root zones, such as vegetables, whose management could benefit from shallower soil water content information. Furthermore, in terms of the accuracy of the data, the errors involved in the soil water content estimation using the AOGAN sensor were found to be in the order of 1 to 5%. Despite the sensor not being able to satisfy the desirable 1% resolution of soil water content data for precision agriculture, suggested by Terry A. Brase [91], it can still be used as a mapping tool for providing comparative assessments of soil water content on large scales. Moreover, accessing surface soil moisture information across large areas and frequently in time allows calibration of evapotranspiration soil models for continuous estimation of soil moisture with depth, valuable for agricultural and hydrological applications (e.g., see [19,27,28,29]). 

Overall, whilst considering the aforementioned limitations, these sensors show promising potential in estimating surface soil water content, with implications in diverse fields including agriculture, bushfire protection management and road construction.

## 7. Conclusions

The non-invasive estimation of soil water content using capacitive-based sensors was investigated in this research. The experimental program entailed testing four new capacitive sensors, the AD7747 (Circular), MPR121 (Rectangular), PCB and AOGAN sensors, against four soil types. Measurements of the dielectric constant of the samples with an Agilent slim form dielectric probe connected to a FieldFox network analyser aided in the comparison of results, analysis and calibration of sensors. The AOGAN sensor was designed and manufactured based on the key advantages of each of the AD7747 (Circular), MPR121 (Rectangular) and PCB sensors. Promising capabilities were observed for the Rectangular and AOGAN sensors, with relatively small errors to estimate the soil water content, particularly for the latter sensor. The effect of soil type on the performance of the sensor was tested by combining data from the samples and it appeared that a single calibration could be adopted to estimate the soil water content. However, adopting a single calibration for all of the four samples resulted in inferior sensor performance compared to when adopting individual calibrations for sand and clay groups separately. Finally, it was demonstrated that the performance of each of the sensors was affected by the level of contact maintained between the sensor and the soil surface and more importantly by the roughness of the soil surface which impacted the soil-sensor contact area. 

## Figures and Tables

**Table 1:** Characterisation of soil samples.

Soil Sample	Location	Clay (%)	Silt (%)	Sand (%)	PL (%)	LL (%)	OM (%)	Salinity (dS/m)	USCS Symbol
**Brighton Group Sand**	Brighton, VIC	<1	2	97	NA	NA	0.08	1.5	SP
**Silty Sand**	Melbourne, VIC	<1	14	85	NA	NA	0.37	2.3	SM
**Silty Clay (Camb)**	Camberwell, VIC	48	42	10	19	28	0.18	3.0	CL
**Clayey Silt (Bun)**	Buninyong, VIC	13	70	17	30	39	0.33	1.6	ML

PL—plastic limit; LL—liquid limit; OM—organic matter; SP—poorly graded sand; SM—fine-grained silty sand; CL—low to medium plasticity silty clay; ML—low to medium plasticity clayey silt.

**Table 2:** Summary of the Circular Sensor and dielectric probe performance against each soil sample.

Soil Sample	Circular Sensor			Dielectric Probe		
	Equation	R^2^	RMSE (%)	Equation	R^2^	RMSE (%)
**Brighton Group Sand**	θ = 0.43C^2.16^	0.71	9.71	θ = 0.057C^2^ + 2.48C − 3.92	0.98	1.08
**Silty Sand**	θ = 2.57C^0.88^	0.67	4.23	θ = −0.052C^2^ + 2.4C − 2.5	0.89	2.46
**Silty Clay (Camb)**	θ = 2.26C^0.96^	0.35	12.38	θ = 0.065C^2^ + 3.19C − 4.5	0.84	4.92
**Clayey Silt (Bun)**	θ = 2.31C^1.12^	0.50	12.15	θ = 0.034C^2^ + 2.34C − 2.34	0.98	1.80

θ = Volumetric Water Content, C = Capacitance (pF), R^2^ = Coefficient of Correlation, RMSE = Root Mean Square Error.

**Table 3:** Cross validation analysis for the Circular sensor for each soil.

Soil Sample	Calibration		Validation	
	R^2^	RMSE (%)	R^2^	RMSE (%)
**Brighton Group Sand**	0.69	7.63	0.30	0.68
**Silty Sand**	0.63	4.25	0.62	4.82
**Silty Clay (Camb)**	0.31	11.82	0.27	13.69
**Clayey Silt (Bun)**	0.44	11.27	0.31	14.48

**Table 4:** Summary of the Rectangular sensor and dielectric probe performance against each soil sample.

Soil Sample	Rectangular Sensor			Dielectric Probe		
	Equation	R^2^	RMSE (%)	Equation	R^2^	RMSE (%)
**Brighton Group Sand**	θ = 0.63C^0.77^	0.96	4.11	θ = 0.057C^2^ + 2.48C − 3.92	0.98	1.08
**Silty Sand**	θ = 1.03C^0.65^	0.96	3.52	θ = −0.025C^2^ + 2.04C − 2.5	0.93	2.86
**Silty Clay (Camb)**	θ = 1.44C^0.65^	0.92	4.95	θ = 0.015C^2^ + 2.09C − 2.5	0.95	4.17
**Clayey Silt (Bun)**	θ = 2.22C^0.60^	0.78	11.22	θ = 0.034C^2^ + 2.34C − 2.34	0.98	1.80

θ = Volumetric Water Content, C = Capacitance (pF), R^2^ = Coefficient of Correlation, RMSE = Root Mean Square Error.

**Table 5:** Cross validation analysis for the Rectangular sensor for each soil.

Soil Sample	Calibration		Validation	
	R^2^	RMSE (%)	R^2^	RMSE (%)
**Brighton Group Sand**	0.96	3.40	0.97	5.35
**Silty Sand**	0.95	3.20	0.95	3.95
**Silty Clay (Camb)**	0.93	4.90	0.93	5.17
**Clayey Silt (Bun)**	0.78	10.32	0.78	14.18

**Table 6:** Summary of the PCB sensor and dielectric probe performance against each soil sample.

Soil Sample	PCB Sensor			Dielectric Probe		
	Equation	R^2^	RMSE (%)	Equation	R^2^	RMSE (%)
**Brighton Group Sand**	θ = 0.007C^2.63C^	0.91	3.96	θ = 0.057C^2^ + 2.48C − 3.92	0.98	1.08
**Silty Sand**	θ = 0.017C^2.40^	0.77	7.10	θ = −0.029C^2^ + 2.11C − 2.5	0.95	2.38
**Silty Clay (Camb)**	θ = 0.003C^3.28^	0.84	9.67	θ = 0.015C^2^ + 2.09C − 2.5	0.95	4.17
**Clayey Silt (Bun)**	θ = 0.008C^2.8^	0.96	3.75	θ = 0.034C^2^ + 2.34C − 2.34	0.98	1.80

θ = Volumetric Water Content, C = Capacitance (pF), R^2^ = Coefficient of Correlation, RMSE = Root Mean Square Error.

**Table 7:** Cross validation analysis for the PCB Sensor for each soil.

Soil Sample	Calibration		Validation	
	R^2^	RMSE (%)	R^2^	RMSE (%)
**Brighton Group Sand**	0.91	3.36	0.91	4.39
**Silty Sand**	0.76	6.72	0.77	7.10
**Silty Clay (Camb)**	0.855	9.44	0.86	9.86
**Clayey Silt (Bun)**	0.96	0.93	0.96	4.09

**Table 8:** Summary of the AOGAN sensor and dielectric probe performance against each soil sample.

Soil Sample	AOGAN Sensor			Dielectric Probe		
	Equation	R^2^	RMSE (%)	Equation	R^2^	RMSE (%)
**Brighton Group Sand**	θ = 0.94C^1.23^	0.91	3.88	θ = 0.057κ’^2^ + 2.48C − 3.92	0.98	1.08
**Silty Sand**	θ = 1.41C^1.15^	0.91	4.8	θ = −0.025C^2^ + 2.04C − 2.5	0.93	2.86
**Silty Clay (Camb)**	θ = 1.23C^1.31^	0.97	2.78	θ = 0.045C^2^ + 2.74C − 2.5	0.88	4.96
**Clayey Silt (Bun)**	θ = 1.49C^1.18^	0.96	4.4	θ = 0.034 C^2^ + 2.34C − 2.34	0.98	1.80

θ = Volumetric Water Content, C = Capacitance (pF), R^2^ = Coefficient of Correlation, RMSE = Root Mean Square Error.

**Table 9:** Cross validation analysis for the AOGAN sensor for each soil.

Soil Sample	Calibration		Validation	
	R^2^	RMSE (%)	R^2^	RMSE (%)
**Brighton Group Sand**	0.89	3.83	0.89	4.66
**Silty Sand**	0.92	4.13	0.92	4.79
**Silty Clay (Camb)**	0.97	2.48	0.97	3.07
**Clayey Silt (Bun)**	0.96	4.02	0.96	4.75

**Table 10:** The response of each sensor against the combined dataset. The response of the dielectric constant data against the corresponding dataset used for each sensor is also shown.

	Sensor			Dielectric Probe		
Sensor	Equation	R^2^	RMSE (%)	Equation	R^2^	RMSE (%)
**Circular**	θ = 2.09C^1.05^	0.53	9.92	θ = 0.029κ’^2^ + 2.21κ’ − 2.5	0.90	3.51
**Rectangular**	θ = 1.31C^0.66^	0.87	7.35	θ = −0.008κ’^2^ + 1.82κ’ − 2	0.95	3.70
**PCB**	θ = 0 006C^2.78^	0.78	11.94	θ = −0.007κ’^2^ + 1.79κ’ − 2	0.95	3.6
**AOGAN**	θ = 1.24C^1.23^	0.92	4.85	θ = −0.023κ’^2^ + 2.09κ’ − 2.5	0.92	3.54

θ = Volumetric Water Content, C = Capacitance (pF), R^2^ = Coefficient of Correlation, RMSE = Root Mean Square Error.

**Table 11:** Performance of the sensors against combined soil, combined sand group and combined clay group data.

	All Soils			Sands			Clays		
Sensor	Equation	R^2^	RMSE (%)	Equation	R^2^	RMSE (%)	Equation	R^2^	RMSE (%)
**Circular**	θ = 2.09C^1.05^	0.53	9.92	θ = 1.99C^1.04^	0.59	5.34	θ = 2.43C^0.99^	0.39	12.36
**Rectangular**	θ = 1.31C^0.66^	0.87	7.35	θ = 0.81C^0.71^	0.95	4.20	θ = 1.82C^0.61^	0.87	6.78
**PCB**	θ = 0 006C^2.78^	0.78	11.94	θ = 0 01C^2.51^	0.81	6.61	θ = 0.004C^3.11^	0.85	11.39
**AOGAN**	θ = 1.24C^1.23^	0.92	4.85	θ = 1.14C^1.21^	0.90	4.95	θ = 1.39C^1.23^	0.96	4.62
